# NK and NKT-Like Cells in Patients with Recurrent Furunculosis

**DOI:** 10.1007/s00005-017-0500-8

**Published:** 2017-12-13

**Authors:** Danuta Nowicka, Ewelina Grywalska, Elżbieta Fitas, Michał Mielnik, Jacek Roliński

**Affiliations:** 1 Department of Dermatology, Venereology and Allergology, Wrocław Medical University, Chalubinskiego 1, 50-368 Wroclaw, Poland; 2 Department of Clinical Immunology and Immunotherapy, Medical University of Lublin, Lublin, Poland

**Keywords:** NK cells, Staphylococcal infection, Staphylococcosis, Recurrent furunculosis, Immune response, Skin infection, In vitro study

## Abstract

To analyze changes in the number and percentage of NK and NKT-like cells in relation to other immune cells as well as to examine associations between increased susceptibility to infections and NK and NKT-like status in patients with recurrent furunculosis (RF) and healthy controls. Thirty patients with RF and 20 healthy age- and sex-matched volunteers were recruited. Blood samples were examined. Lymphocyte count and cytometric analyses were conducted. For statistical analysis, the Student’s *t* test, *F* test, and Brown–Forsythe test were used for comparison between groups of variables. Associations were assessed with Pearson coefficient. Patients with RF had lower lymphocyte count than controls. Additionally, they presented with the following changes in the blood picture: a significant increase in the number of NK cells with a CD3^+^CD16^+^CD56^+^ phenotype; a proportional increase in the number and percentage of NKT-like cells with a CD3^+^CD16^+^CD56^+^ phenotype; a significant decrease in the number and percentage of T CD3^+^ cells. The number of NK cells was strongly positively correlated with the number of CD3 cells (*r* = 0.6162). The number of NKT cells was strongly positively correlated with CD3 cells (*r* = 0.6885) and CD3CD8 cells (*r* = 0.5465). Periodic exacerbations in RF are associated with the development of furuncles, which are a result of many already discovered as well as just being examined mechanisms. One of them is a significant increase in the number and most likely activation of NK and NKT-like cells during the formation of the inflammatory process and furuncles.

## Introduction

Furuncles and furunculosis are the most common skin infections in dermatogical practice. *Staphylococcus aureus* is the causative agent of most of them. Under certain conditions, the environment may change and permit bacteria to penetrate into the hair follicles, proliferate, and induce intensive inflammatory reaction which at first is located only at the opening of the hair follicle, but next, it spreads to all the structures of the hair follicle and adjacent tissues (perifolliculitis). Toxic substances produced by *S. aureus* contribute to the development of foci of necrotic tissues and after their healing—scars. Infection may have a fulminant course especially in patients infected by human immunodeficiency virus (HIV), patients suffering from diabetes, or those being treated with immunosuppressant drugs. Also, in patients with impaired functioning and structure of the skin barrier which is characteristic for patients with atopic dermatitis, recurrent or severe furuncles are observed. Despite the frequent occurrence, factors that determine susceptibility to furuncles and their recurrence in immunocompetent people without atopy and metabolic diseases have not yet been examined.

Natural killer (NK) cells are a part of the lymphatic system. They are produced in the bone marrow where they enter into the circulation and migrate to the liver, spleen, lungs, and many other organs. The presence of receptors for many chemokines on their surface allows them to migrate to various inflamed locations (Kapetanovic and Cavaillon 2007; Pesce et al. 2016; Shi et al. 2011). In the peripheral blood, the mean percentage of NK cells accounts for about 10%. They have a similar morphologic structure like that of the lymphocytes, but they have small granules in their cytoplasm. These granules are of lytic character and contain perforins—molecules that bind to membranes, and death-inducing granzymes. The presence of a cluster of differentiation (CD) 16 antigens, which serves as a IgG Fc receptor on the NK cells, enables the lysis of cells possessing IgG on their surface via antibody-dependent cell-mediated cytotoxicity. Human NK cells express many receptors which belong to the killer cell immunoglobulin-like receptor family. For many years, NK cells were regarded responsible only for non-specific cellular immunity involved in the antiviral and anticancer defense. Still, their particular importance reflects the fight with viral infections and cancer diseases. Also, rare immune deficiencies manifest themselves in various viral diseases (Orange 2013). Increasingly, researchers emphasize the role of NK cells in the antibacterial defense in various locations in the organisms such as the mucosa of digestive, respiratory, and reproductive system as well as joints or even nervous system (Ivanova et al. 2014; Shi et al. 2011).

The aim of the study is to analyze changes in NK and NKT-like cells in relation to other immune cells in patients with recurrent furunculosis as well as to evaluate the relationship between increased susceptibility to infections and NK and NKT-like status in this group of patients in comparison with healthy controls.

## Materials and Methods

### Study and Control Groups

For the study group, 30 patients (15 men and 15 women) with a diagnosis of recurrent furunculosis were recruited at the Outpatient Immunology Clinic of the Medical University of Lublin between 2014 and 2017. The control group included 20 healthy age- and sex-matched volunteers.

None of the patients and controls had been receiving drugs affecting the immune system including immunosuppressive or immunomodulative treatment; none had showed any signs of infection (at least 1 month before the study), autoimmunity, neoplastic or allergic disease. None had received blood transfusions.

The peripheral blood samples were drawn from the basilic vein for the frequencies of selected lymphocyte subsets (5 mL of peripheral blood was collected into tubes with the anticoagulant EDTA). Percentages of lymphocyte subsets were assessed on fresh peripheral blood samples from the study group and controls. This study was carried out in accordance with the Code of Ethics of the World Medical Association (Declaration of Helsinki) for experiments involving humans. The Local Ethical Committee at the Medical University of Lublin approved the research. All the patients gave their written informed consent prior to entering the study.

### Immunophenotyping of Peripheral Blood Cells

Samples for cytometric analyses were prepared from freshly obtained peripheral blood incubated with a set of monoclonal antibodies: anti-CD3 FITC, anti-CD3 PECy5, anti-CD4 FITC, anti-CD8 PE, anti-CD19 PE, anti-iNKT FITC, anti-CD25 PECy5, and anti-CD3 FITC/anti-CD16 PE/anti-CD56PE (BD Pharmingen, USA). The samples were deprived of erythrocytes by adding lysing solution (FACS Lysing Solution, Becton Dickinson, USA). The immunophenotype of peripheral blood cells was determined with a FACSCalibur flow cytometer (Becton Dickinson, USA) equipped with an argon laser emitting at 488 nm. The results were analyzed with CellQuest Pro software (Becton Dickinson, USA).

Statistical analysis was carried out with the R Project for Statistical Computing v. 3.4. Descriptive statistics were calculated. The Student’s *t* test, *F* test, and Brown–Forsythe test were used for comparison between groups of variables. Associations between percentages and numbers of NK, NKT, CD3, and CD3CD8 cells in patients with recurrent furunculosis were assessed with Pearson coefficient for dependent variables. The criteria for statistical significance were set at *p* < 0.05.

## Results

**Fig. 1:**
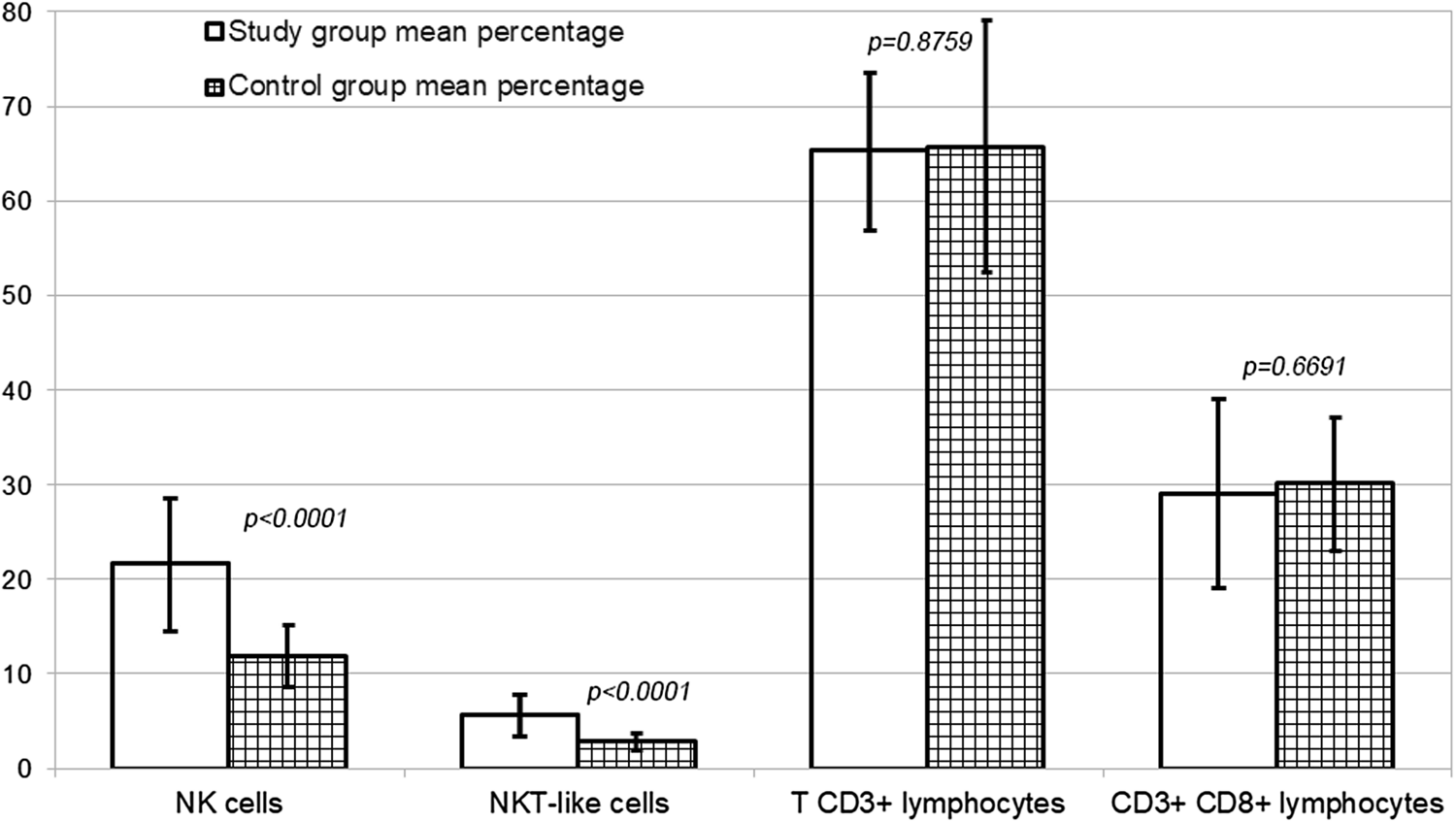
Comparison of the mean percentage of cells between the study group and control group

The study group included 30 patients aged between 19 and 44 years (average 31.1 years). Recurrent furunculosis started at the average age of 27.7 years (range 17–42 years). The duration of the disease varied 1–7 years. Remission period lasted 4–20 years. Patients received 4–20 antibiotic therapy courses and underwent 4–12 surgeries. The control group included 20 healthy, age- and sex-matched volunteers with the mean age of 31.95 ± 4.1 years. Results of the cytometry of peripheral blood mononuclear cells are presented in Table 1 and Fig. 1.

**Table 1:** Comparison of the mean number of cells (G/l) counted with the cytometry of peripheral blood mononuclear cells between the study group and control group

Parameter	Study group (mean ± SD)	Control group (mean ± SD)	*p* value
Lymphocytosis	1.9768 ± 0.5601	2.1750 ± 0.5388	**0.0397**
NK cells	0.3943 ± 0.1758	0.2609 ± 0.1080	**0.0039**
NKT-like cells	0.0693 ± 0.0359	0.0422 ± 0.0057	**0.0037**
T CD3^+^ lymphocytes	1.2024 ± 0.3772	1.4553 ± 0.1929	**0.0457**
CD3^+^CD8^+^ lymphocytes	0.3581 ± 0.1887	0.4528 ± 0.0114	0.0756

Significant difference marked in bold

*SD* standard deviation

Patients with recurrent furunculosis had a lower lymphocyte count than patients from the control group. Among lymphocytes, a significant increase in the number of NK cells with a CD3^+^CD16^+^CD56^+^ phenotype was observed; its percentage increased almost twofold. Similar to NK cells, the number and percentage of NKT-like cells with a CD3^+^CD16^+^CD56^+^ phenotype also increased proportionally. On the other hand, the number and percentage of T CD3^+^ decreased significantly in the group with furuncles. Additionally, a trend towards a decrease in the number and percentage of CD3^+^CD8^+^ cytotoxic lymphocytes was visible, however, without a significant difference in comparison to the control group.

The analysis of associations between the percentage (%) of NK, NKT, CD3, and CD3CD8 cells in patients with recurrent furunculosis showed very weak correlations. The analysis of associations between the number of examined cells (G/l) revealed weak positive correlation between NK and NKT cells (*r* = 0.3071), strong positive between NK and CD3 cells (*r* = 0.6162), and very weak between NK and CD3CD8 (*r* = 0.2316). The number of NKT cells was strongly positively correlated with the number of CD3 cells (*r* = 0.6885) and CD3CD8 cells (*r* = 0.5465).

## Discussion

A significant decrease in the lymphocyte count in patients with recurrent furunculosis in comparison with healthy controls undoubtedly results from the characteristics of bacterial inflammatory process. In this process, a stimulation of neutrophils and monocytes/macrophages in the bone marrow takes place as a result of cellular response to bacterial pathogens leading to the development of considerable predominance of neutrophils and monocytes in the blood picture. NK cells are one of the fastest responders to inflammation. Their activity can be observed already after 4–6 h after exposure to bacterial pathogen (Ślebioda et al. 2012). During bacterial inflammation, the stimulation of NK cells by dendritic cells and macrophages is necessary for the activation of their cytotoxic capacity (Hall et al. 2013; Horowitz et al. 2011). In the optimal immune response, there is a strict relationship between NK cells and dendritic cells. Interferon γ produced by NK cells promote activation of dendritic cells, which promote activation of NK cells via secretion of interleukin (IL)-15, IL-12, and IL-18 and as a result, increase their proliferation and cytotoxic capacity (Thomas and Yang 2016). Johanson et al. (2016) examined an in vitro culture of peripheral blood mononuclear cells and revealed that *S. aureus* 161:2 induced activation of CD4CD8, and NK cells. Hall et al. (2013) reported that in mice experimentally infected with *Citrobacter rodentium* to cause colitis, depletion of NK cells led to higher bacterial loads, but less severe colonic inflammation because of a reduction in pro-inflammatory cytokines produced by these cells. Therefore, the increase in the number of NK cells protects against disseminated infection, while locally, it produces exacerbation of inflammation symptoms (Hall et al. 2013).

In patients with recurrent furunculosis, a significant increase both in the percentage and number of NK cells was observed in comparison with the values obtained in the control group. This demonstrates their important role in the fight against *S. aureus* infection occurring in this disease. Apart from the elevation of the percentage and number of NK cells, an increase in the number of NKT-like cells was observed; however, NKT-like cells count does not always change in bacterial infections. Their presence was not confirmed neither in the lumen of pulmonary alveoli nor in the lung tissue during infections by *S. aureus* as well as in some models of pulmonary infection caused by mycobacteria and *Pseudomonas*. Also, an increase in the number of CD8 cells in the lumen of pulmonary alveoli and the number of T CD8 cells in the lung tissue was not found (Small et al. 2008). In patients with recurrent furunculosis, such changes in the blood picture may be a result of prolonged exposure to pathogen in which T CD3^+^ cells become involved rather than less mature NK cells as well as properties of *S. aureus* and its toxins in engaging specific immunity response. It is interesting that in our study, the percentage and number of T CD3^+^ are significantly lower in the study group than in the control group which may express the pathology occurring in this disease or be related to the development of other type of cells involved in cytotoxic processes but originating from them. CD3^+^CD16^+^CD56^+^, called NKT-like cells, belong to such type of cells. Keratinocytes may be engaged in the process of their production, because there are many of them in the skin—the organ in which furuncles develop. Inflammatory process is located in the skin, but changes in this organ may have an effect on the composition of the blood cells including occurrence of various phenotypes of NK cells and degree of maturity depending on the organ affected by inflammation (Sharma and Das 2014). However, it may be the other way around; changes in the blood picture may be responsible for the development of inflammatory process in the skin. These possibilities are associated with different routes of infection by *S. aureus* and are examined in the number of papers on recurrent furunculosis which include factors such as reduced immunity, poor personal hygiene, or impaired skin barrier due to concomitant skin diseases (El-Gilany and Fathy 2009; Ibler and Kromann 2014). Until present, phenotypes of skin NK cells has been examined in melanoma, psoriasis, and allergic contact dermatitis (Sharma and Das 2014). Studies on NK and NKT cells in bacterial infections including recurrent furunculosis have not been found.

An increase in the number of NK cells contributes to the impairment in the immune system leading to the increase in the number of cells involved in the inflammation and all of its consequences. Zhou et al. (2013) showed that during severe influenza virus infection of the lungs, NK cells contribute to increased mortality in mice. The most recent reports demonstrate that subpopulations of NK cells play various roles during viral infections; they may function as classical effectors or stimulators of adaptive immunity helping to prime immune responses (Zamora et al. 2017). As Small et al. (2008) suggest, the role of NK cells in the antibacterial defense cannot be generalized in relation to various pathogens, route of infection, or tissues of infected organ. It requires pathogen- and organ-specific research and interpretation (Small et al. 2008).

In conclusion, periodic exacerbations in recurrent furunculosis are associated with periodic development of boils which are a result of many already discovered as well as just being examined mechanisms. One of them is a significant increase in the number and most likely activation of NK and NKT-like cells at the formation of the inflammatory process in the form of recurrent furuncles.

## Abbreviations

CD: Cluster of differentiation
HIV: Human immunodeficiency virus
IL: Interleukin
NK: Natural killer cells
NKT-like: Natural killer T-like cells
